# Lymphatic Senescence: Current Updates and Perspectives

**DOI:** 10.3390/biology10040293

**Published:** 2021-04-03

**Authors:** Sebastian Lucio Filelfi, Alberto Onorato, Bianca Brix, Nandu Goswami

**Affiliations:** 1 Physiology Division, Otto Loewi Research Center, Medical University of Graz, 8036 Graz, Austria; sebastian.filelfi@stud.medunigraz.at (S.L.F.); bianca.brix@medunigraz.at (B.B.); 2 Oncology Reference Centre, Institute of Hospitalization and Care with Scientific Characterization, 33081 Aviano, Italy; a.onorato@linfamed.it; 3 Department of Health Sciences, Alma Mater Europeae Maribor, 2000 Maribor, Slovenia

**Keywords:** aging, lymphatics, lymphatic vasculature

## Abstract

**Simple Summary:**

The lymphatic system is involved in tissue homeostasis, immune processes as well as transport of lipids, proteins and pathogens. Aging affects all physiological systems. However, it is not well studied how aging affects the lymphatic vasculature. Therefore, this review aims at investigating how senescence could lead to changes in the structure and function of the lymphatic vessels. We report that lymphatic senescence is associated with alterations in lymphatic muscles and nerve fibers, lymphatic endothelial cells membrane dysfunction, as well as changes in lymphatic pump, acute inflammation responses and immune function.

**Abstract:**

Lymphatic flow is necessary for maintenance of vital physiological functions in humans and animals. To carry out optimal lymphatic flow, adequate contractile activity of the lymphatic collectors is necessary. Like in all body systems, aging has also an effect on the lymphatic system. However, limited knowledge is available on how aging directly affects the lymphatic system anatomy, physiology and function. We investigated how senescence leads to alterations in morphology and function of the lymphatic vessels. We used the strategy of a review to summarize the scientific literature of studies that have been published in the area of lymphatic senescence. Searches were carried out on PubMed and Web of Science using predefined search queries. We obtained an initial set of 1060 publications. They were filtered to 114 publications based on strict inclusion and exclusion criteria. Finally, the most appropriate 57 studies that specifically addressed lymphatic senescence have been selected for the preparation of this review. Analysis of the literature showed that lymphatic senescence is associated with alterations in lymphatic muscles and nerve fibers, lymphatic glycocalyx function of lymphatic endothelial cells, effects of chronic ultraviolet light exposure and oxidative stress as well as changes in lymphatic pump, acute inflammation responses and immune function. The current review underscores the relevance of the understudied area of lymphatic senescence. Continued research on the impact of aging on the structure and function of the lymphatic vasculature is needed to provide further insights to develop innovative clinical diagnostic—and treatment—modalities as well as to reduce the morbidity associated with diseases related to the lymphatic system.

## 1. Introduction

An optimal and functioning lymphatic transport system is necessary to transport lipids absorbed in the gut, immune cells and to maintain fluid and macromolecular homeostasis [1,2]. Therefore, the lymphatic system is involved in several physiological processes, which has been reviewed elsewhere [3]. The lymphatic system, through direct and indirect mechanisms, is involved in immune responses. Malfunction of the flow and lymphatic vessels can, for example, be a contributing factor in the development of inflammatory disorders, metabolic disorders including obesity, immune intolerance, cardiovascular diseases, as well as septic shock and infectious diseases [3]. There exist two types of driving forces that influence lymphatic flow, distinguished by the difference in contraction of lymphangions (functional unit of the lymphatic vessels). These forces are further classified into active/intrinsic (action capacity generated by contractions of the muscles located near the valves of the lymphangions) and passive/extrinsic (made up of all other forces that significantly influence the lymphatic flow) [4].

Lymphatic function can be influenced by intrinsic and extrinsic factors, including active and passive lymph pumps and hydrodynamic factors like shear and pressure [5]. The lymphatic system is involved in the transport of fluid from tissue interstitial spaces. The lymphatic vessels move fluids and different compounds (such as immune cells, macromolecules and lipids) from the interstitium to larger veins [1]. Therefore, lymphatic transport is a fundamental component of the processes such as lipid absorption, management of body fluids, immune function and macromolecular homeostasis [6]. The transport of lymphatic fluid is influenced by the intrinsic pump, which, in turn, depends on the contractile capacity of the collectors of the larger lymphatic vessels that are capable of transporting the lymphatic fluid against a pressure gradient [7]. The extrinsic forces influence the lymphatic vessels passively and differentially, based on the anatomical position of these vessels. These external factors are represented by force of gravity, muscle contractions, arteries pulsations and respiratory activity, for example.

Aging has been associated with alterations in muscle strength and function (e.g., sarcopenia), with changes in the cardiovascular response to perturbations (increased risk of orthostatic intolerance and falls) as well as changes in bone tissue, upon others [8,9]. Those physiological changes due to aging can be observed in all organ systems [8,10]. However, limited knowledge is available on the effects of aging on the lymphatic vasculature structure as well as function. Therefore, the current review intends to provide a collective update on the scientific studies from literature related to lymphatic aging/senescence. Presented here are studies from animal models as well as from clinical data. Underlying pathways through which aging may impact the structure and/or function of the lymphatic vasculature are addressed.

## 2. Search Methodology

**Figure 1:**
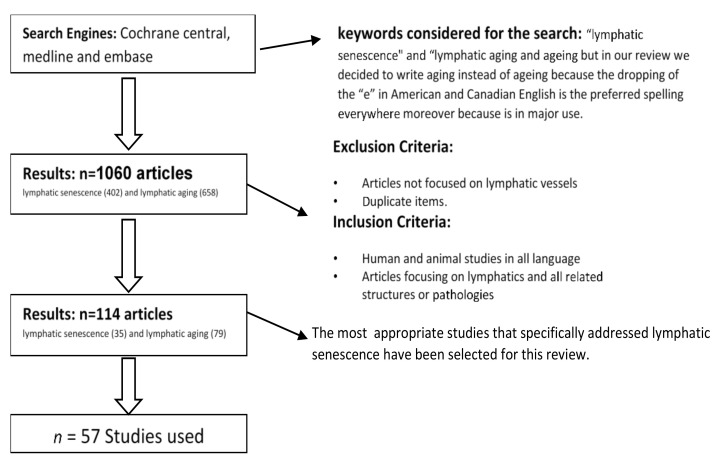
Flowchart of the literature selection process. The 57 most appropriate studies that specifically addressed lymphatic senescence have been included.

The current literature on the subject of “lymphatic senescence” was systematically reviewed and summarized in a review. Both primary and secondary sources of literature on the topic were examined. The systematic approach for the current review is shown in Figure 1.

PubMed and Web of Science served as search engines and were used to access relevant literature. The first approach in the search of this thematic yielded in a total number of 1060 articles. Articles available in English language and articles focusing on the lymphatic system were included. Different combinations of following MESH terms were used when formulating the search: “lymphatic senescence” and “lymphatic aging”.

## 3. Reporting Structure of the Results

The initial search provided 1060 articles entries on “lymphatic senescence” (402) and “lymphatic aging” (658). After application of inclusion/exclusion criteria, a total number of 114 studies (35 on lymphatic senescence and 79 on lymphatic aging) were further considered. Included were human and animal studies as well as articles focusing on lymphatics and all related structures or pathologies. Excluded were studies not focusing on the lymphatic vessels and duplicates. Finally, the most appropriate 57 studies that specifically addressed lymphatic senescence have been selected for the preparation of this review. The research articles were categorized and discussed in specific sections as follows:

Section 4.1: Aging of the lymphatic vessels
Section 4.2: Aging of the lymph nodes
Section 4.3: Lymphatic senescence and lymphatic muscles
Section 4.4: Lymphatic vessel and nerve fibers across aging
Section 4.5: Senescence and alteration in glycocalyx function in lymphatics vessels
Section 4.6: Physiology of the lymphatic pump
Section 4.7: Effects of chronic ultraviolet light exposure on lymphatic dysfunction
Section 4.8: Aging induced oxidative changes in lymphatic vessels
Section 4.9: Aging-induced acute inflammation changes and their effects on lymphatic vessels and perilymphatic tissues
Section 4.10: Lymphatic senescence and immune function
Section 4.11: Lymphatic senescence and clinical outlook.

## 4. Detailed Analysis of the Included Literature

Different types of animals and different breeds have been used in the selected articles. About 42% of these animals were rats of which 37.5% of Fisher-344 rats (in 75% they studied mesenteric lymphatic vessels and in 8.3% cerebrospinal fluid), 12.5% C57Bl/6 mice (in 25% they studied mesenteric lymphatic vessels), 9.4% Wistar rats (in 100% they studied mesenteric lymph duct), 6.2% Sprague-Dawley rats (in 50% they studied intestinal lymphatic channel) and 3.1% BALB/c mice.

About 3% of the studies were carried out on humans, in the following tissues: femoral regions, vocal folds and drainage larynx, cervical, auxiliary, inguinal, popliteal, mesenteric, capillaries, nasal and cerebral lymphatic vessels, lymphatic intact skin, lymph supply of the knee menisci, interstitial lymphatics fluid traffic and endothelial lymphatic vessels.

### 4.1. Aging of the Lymphatic Vessels

When discussing aspects related to aging, it is important to distinguish between the effects of biological aging and that due to environmental factors [11]. Dermal changes involve a reduction of mast cells, macrophages, fibroblasts and collagen and elastic fibers, it also involves the dilatation of the lymphatic channels. Human aging is characterized by a series of disorders that affect all tissues. For example, dilation of the lymphatic channels occurs in aged skin [11]. To a certain extent, dilation also occurs following an elastosis, regardless of age, also leading to a dilation of the superficial capillaries [11].

As aging progresses, a decrease in lymphatic vessel function and lymphatic flow is observed, arising due to a significant reduction in pumping rates, including pump frequency, amplitude, and fractional lymph flow [12]. In the resting phase, these changes of the lymphatic collectors [13], can generate a low level of tissue edema. Analysis of thoracic duct segments in elderly rats showed that lymphatic tone was lower and accompanied by decreased amplitude of lymphatic contraction [12]. This may be an indicator of weakening of muscle cells, which occurs due to aging, causing a reduced ability to generate sufficient force to maintain the appropriate level of contractile tone and force in the lymphatic vessel.

Furthermore, it has been shown that aging alters the glycocalyx (and consequently the permeability of the lymphatic collectors), lymphatic contractility and reduces junctional proteins [7]. It is important to understand the mechanisms by which nitric oxide (NO), reactive nitrogen species (RNS) and reactive oxygen species (ROS) may interrupt the lymphatic contraction. This is important when NO/ROS concentrations are high, as it occurs in chronic inflammation. Moreover, lymphatic function can be affected by metabolites released into the aged tissue microenvironment [14].

A decrease in the function of the lymphatic pump associated with aging in the mesenteric lymphatic vessels (MLV) has also been observed. This suggests that aging can significantly affect the lymphatic muscles [7]. For instance, measurements of the amount of muscle fibers in the MLVs near the lymphatic valve showed that upstream of the lymphatic valves, there is a low density of muscle fibers in aged lymphatic vessels. In contrast, downstream of the lymphatic valve a high density of muscle fibers was observed. These changes are believed to modify the biomechanics of the gating of the lymphatic valve and/or the electrical coupling between lymphangions, causing a limitation of adaptive reserves in the elderly lymphatic vessels [7].

### 4.2. Aging of the Lymph Nodes

In aged lymph nodes, decreases in the total number of double-negative (podoplanin-CD31) cells as well as a disorganization in their distribution has been reported. Podoplanin and CD31 are expressed in the lymphatic vessels and are used for immunohistochemical staining of the endothelial subtype [15]. The structural disorganization observed in aging lymph nodes could be responsible for the decreases in T lymphocytes and this could lead to a decrease in the immune response [15]. Older age was associated with a reduction in skin lymphatic density. Similar results have been obtained in human photoaged skin [16]. Finally, a reduced complexity of the lymphatic network was found due to a reduced branching of the lymphatic tree in elderly mice, demonstrating a process of involution of cutaneous LVs in aging [17].

The senile involution seen in lymph nodes of humans affects the structure of the lymph node. Moreover, this lymph node degeneration process is progressive [18]. In the study it was found that nonoperating lymph nodes contained coils of lymphatics, with no cortex or medulla, and little fibrous connective tissue on histopathology. This information explains clinical conditions in the elderly, in particular their poor ability to fight infections and prevent the development of tumor metastases [18].

### 4.3. Lymphatic Senescence and Lymphatic Muscles

The musculature of the thoracic duct reaches its maximum size around 30 years of age [19]. In eldery people, atrophy of the lymphatic muscles and a decrease in the elastic lymphatic matrix is seen, which leads to sclerosis of the thoracic duct. Appearance of similar formations in other lymphatic vessels also occur (e.g., aneurysms near the lymphatic valves). Additionally, it has been reported that the number of connections between lymphatic vessels in the human mesentery decrease considerably with age [19].

The lymphatic muscle is composed by a mixed type of myosin, resembling both smooth and striated muscle myosin. Recent studies have shown that it is actually a new class of muscle, which is quite distinct from cardiac and smooth muscles. Lymphatic muscles are also modulated by mechanical, neuromodulatory and vasoactive factors. The peculiarity of the lymphatic muscle lies in the fact that there are Ca^2+^ type-T and type-L channels (characteristic of smooth muscle cells and involved in both cardiac and lymphatic tissues) [7]. Up to 93% of the length of the lymphatic vessel is made up of a circular oriented musculature around the post valvular areas, consequently providing the greatest lymphatic contractile force [20]. With regards to the longitudinally oriented muscle cells that connect the pre-valvular area and the post-valvular area from the corresponding ones, they have been reported to have a lower muscular investiture in the lymphatic wall. Due to their shape, it is thought that during lymphatic contraction these muscles shorten longitudinally leading to a displacement, which brings their positions closer during lymphangion systole. It is believed that during aging these functions of the lymphatic muscles are altered because there is a decrease in the investiture of these muscle cells in the pre-valvular and valvular areas. Therefore, it is hypothesized that this is one of the main causes of a decreased ability of the lymphatic vessels to adapt adequately to contractility in the various preload/afterload alterations. Finally, there may be a risk that thin-walled areas in aging MLVs low in muscle can be transformed into aneurysm-like formations, which were previously referred to as “varicose bulges”. These aneurysm-like formations can create low-speed turbulent lymphatic flow and lead to the accumulation of pathogens, cancer cells and various cells, which can subsequently pass through the thin endothelial cell layer.

Functionally, aging-associated changes in lymphatic muscle cells can lead to periods of increased functional demands required to contract the lymphatic vessels (which occur periodically in lymphatic networks). In summary, decrease in the amplitude of lymphatic contraction, along with the lowering of lymphatic tone in the segments of the thoracic duct, are seen in the aged thoracic lymphatic vessels, even at low pressure. Additionally, it has been observed that as the pressure increases, magnitude of the lymphatic contraction decreases to a greater extent in older lymphatic vessels. This negative inotropy is also accompanied by a decrease in the frequency of contraction, especially at high transmural pressure levels (5 cm H_2_O) [20]. Similar findings are seen in age-related negative chronotropy in the thoracic duct that leads to a decrease in the lymphatic pump flow and accentuating the reduced pumping capacity of the aged thoracic duct [12]. It has also been shown that the aged thoracic duct has a poor ability to adapt its contractility following an increase in transmural pressure (until 5 cm H_2_O), which may lead to an insufficient or total absence of fluid transport [12].

Another consequent cause of the loss of muscle efficiency is a lower efficacy of the lymphatic valve closure causing a consequent reflux of lymph into the elderly lymphatic collecting vessels [21]. It has been reported that the ability of lymphatic collectors to transport pathogens in the elderly is compromised [13]. Consequently, pathogens can spread in the opposite direction to the lymphatic flow due to the possible reflux caused by the lymphatic valves [20].

Aging is associated with a reduction in the levels of proteins, which regulate muscle contraction [13]. The contractile proteins myosin and troponin, myosin binding proteins and those associated with the cytoskeleton (dynein, actin and gelsolin) in elderly mice have been shown to be reduced in lymphatic collectors. Other proteins which are known to be involved in the generation of the muscle cell action potential have also been reported to be decreased in the lymphatic collector of aged mice [13].

Administration of 100 µM L-NAME into aged mesenteric lymphatics in rats showed that active pumping was severely decreased, mainly due to the associated decrease in contractile speed in aged lymphatics. These changes are believed to be related to the enlargement of the MLVs with the aging process. At the same time, pumping of aged MLVs can be restored to pumping levels of adult vessels by increasing the rate of contraction induced by NO elimination [12]. NO modulates an ongoing contraction in both, a localized and coordinated manner along the main lymphatic vessel to increase shear forces and any NO-dependent vasoactive mediators in the lymph or tissues. NO is produced in the lymphatic endothelial cell layer by endothelial nitric oxide synthase (eNOS) in response to flow or shear stress. Studies state that aging weakens the contractility of MLVs by limiting the ability of these vessels [22,23]. During the phasic contraction, both the tubular section and the lymphatic valve section increase their NO generation. By inducing vasorelaxation, NO leads to the reduction of vascular shear force while lymphatic flow is increased. The accumulated NO generated is able to increase the frequency of lymphatic vessels contraction. Baseline NO plays important role in maintenance of contractile diameter to modulate lymphatic flow. The phosphorylation of 20 kDa (kilodalton) myosin light chain (MLC20) plays an important role in smooth muscle contraction and is also considered a critical determinant of tonic lymphatic contraction.

Inflammation can increase lymphatic flow and is also accompanied by increases the volume of interstitial fluid, which arises due to increased microvascular permeability. The increase in volume induces a stretch on the lymphatic vessel wall, increasing the frequency and/or the contractile force. On the other hand, decreases in lymphatic drainage occurs due to synthesis of certain neuromediators linked to inflammatory and immune responses. These include substance P, the peptide related to the calcitonin gene (CGRP), neuropeptide Y, prostaglandins and vasoactive intestinal polypeptide. It is believed that all of these substances affect the contractile function of the lymphatic vessels.

Aging introduces significant changes in the modulation of contractility of the lymphatic wall related to the functional increase of histamine as an endothelium-derived relaxation factor in MLVs and the simultaneous elimination of NO [24]. Increase in histidine decarboxylase, an enzyme capable of producing histamine in aged MLVs, was also seen. Similarly, the transmural stretch pressure-dependent regulatory reactions of contracting MLVs are not affected by intralymphatic histamine [24]. This is not true for the effects of histamine released from sources external to the lymphatic vessels on lymphatic contractility [24]. Overall, the available literature suggests that histamine is a strong modulator of lymphatic contractility [25]. At lower concentrations, histamine stimulates lymphatic contractility, while at higher concentrations, histamine induces relaxation and contractile inhibition [25].

### 4.4. Lymphatic Vessel and Nerve Fibres across Aging

The lymphatic system exhibits a rich innervation with many different neuropeptides and neurotransmitters. The presence of sympathetic and parasympathetic nerve systems on lymph vessels has been reported. These innervations play important roles in the maintenance—and regulation—of the hydrodynamic forces and humoral factors that are important regulators of lymphatic functions. Furthermore, the innervation of the lymphatic vessels is not the same throughout the body. In the mesenteric and femoral lymphatic vessels, there is a greater distribution of nerve fibers than in the cervical region, thus allowing better lymphatic flow from the lower regions of the body [26]. In addition, the formation of nitric oxide plays an important role in regulating lymphatic pumping and the hydrodynamic factors involved in lymphatic pump function [19]. Substance P was reported to have a positive chronotropic and inotropic effect in lymphatic vessels thereby improving pump operation [27].

A change in the functions of these nerve fibers and neurotransmitters can negatively affect the physiological homeostasis of the organism. Moreover in this context, aging-related changes play a key role. Aging is associated with a reduction of all specific nerve fibers, thus influencing the lymphatic function and flow regulation, which can lead to the development of lymphatic pathologies and/or aggravate existing pathologies in the lymphatic system [26].

### 4.5. Senescence and Alteration in Glycocalyx Function in Lymphatics Vessels

The glycocalyx acts as a barrier between lymphatic fluid and the endothelium thus decreasing the permeability of the lymphatic collectors and prevents pathogens and immune cells from adhering to the endothelium [28]. In aged rats, decreases in glycocalyx, reduction in thickness and destruction in the continuity of the lymphatic endothelial membranes was seen [13]. Zolla et al (2015) used ultrastructural, biochemical, and proteomic analysis of rat tissue samples and observed differences in the composition of the glycocalyx between adult and elderly animals [13]. As a result, pathogens may more easily escape from lymphatic collectors into the surrounding tissue. The accompanying increased leakage of lymphatic fluid and immune cells leads to a greater risk of infections and inflammation in older rats. Finally, several glycocalyx-associated proteins were downregulated in older MLVs as compared to younger ones [29].

### 4.6. Physiology of the Lymphatic Pump

Lymphatic vessels can display a coordinated contraction that can propagate in the opposite direction to the flow [21]. After the contraction of the muscle complex of the lymphatic vessel there is a “diastolic” fear leading to relaxation before the next contraction takes place. In the muscle collectors of the lymphatic vessels (except diaphragmatic lymphatic vessels), the contraction is activated by the distension of the lymphatic wall. By doing so, the lymphatic inotropy will stabilize as the chronotropy continues to rise. As pressure and stretch increase, the inotropy will decrease and thus the lymphatic pump will begin to fail and the chronotropy will reach its maximum level [19]. Peripherally, smaller lymphatics have been found to have a peak pumping activity at higher transmural pressure [19]. This suggests that the latter can generate higher pressures to overcome the high resistance to outflow due to their position in the lymphatic network [19]. In the aforementioned study, they studied phasically active and non-active segments of the lymphatic thoracic duct separately, highlighting that only in the active one there were differences in the flow and shear of the lymphatic pump function. Because of this, it has been hypothesized that phasic pumping is inhibited when the axial flow gradient exceeds a certain threshold, which allows (due to the increase in pressure) the lymphatic flow to be guided through the lymphatic vessel. Thus, if the pressure increases, it does not require phasic contractions [19]. It is important to state that the structure of the vessels changes depending on its position: the segments of the initial lymphatic depend on a strong pump to overcome the high resistance to outflow, while the lymphatic collectors are not as strong as a pump and they function more like conduits [30]. The pumping/resistance function of the lymphatic vessels is coordinated by a continuous regulation of intrinsic and extrinsic flows to adapt to local transport needs [19]. That is, at low levels of extrinsic-force generated lymphatic flow, intrinsic pump influences prevail, with periodic release of NO due to phasic flow/lymphatic pump shear patterns, which maintain functional lymphatic transport. On the other hand, when the extrinsic-force generated lymphatic increases, the influences of the extrinsic forces are dominating, thus leading to increases in the release of NO and inhibition of intrinsic pumping [19].

### 4.7. Effects of Chronic Ultraviolet Light Exposure on Lymphatic Dysfunction

Lymphatic vessels have been shown to play an important role in skin inflammation and in the skin response to ultraviolet B (UVB) ray exposure [31]. Chronic exposure to UVB leads to the degradation of the elastic fibers probably induced by a reduced adhesion from the extracellular matrix to the lymphatic endothelial wall. Therefore, UVB exposure could induce changes in the lymphatic vessels by influencing the elastic fibers and interstitial collagen [32]. The question arises whether UVB rays are capable of inducing lymphatic dysfunction. It has been observed that following exposure of UVB rays on the epidermis, increases in vascular endothelial growth factor A (VEGF-A) occur. The latter derived from the epidermis promotes the enlargement and loss of functions of the lymphatic vessels. This was confirmed in studies carried out on transgenic mice with overexpression of VEGF-A exposed to acute UVB irradiation. These animals showed enlarged lymphatics and pronounced skin edema [33]. On the other hand, treatment with a VEGF-A blocker prevented UVB-induced enlargement of the lymphatic vessels [33], suggesting that the same VEGF-A molecule plays important roles in vascular hyper-permeability, lymphatic flow and consequently, reduced lymphatic drainage. Structurally, cell-cell adherent junctions of lymphatic endothelial cells have the function of maintaining the physiological and anatomical characteristics of the lymphatic vessels. Acute UVB irradiation exposure on the skin causes a significant dilation of the lymphatic vessels, and therefore modifies their structure leading to changes in their function as well as lymphatic vascular hyperpermeability [34]. This means that UVB irradiation on the skin causes a dysfunction of the lymphatic function [35].

Apelin, a bioactive peptide, has been shown to be important in the maintenance of the lymphatic vascular system as it plays a key role in containing UVB-induced skin inflammation [36]. Overexpression of apelin in the epidermis in mice prevented enlargement and hyperpermeability of blood vessels and lymphatic vessels following UVB irradiation [37]. Additionally, administration of a VEGF-A blocker prevented the dilation of the lymphatic vessels induced by UVB exposure [33], thus further supporting the evidence related to the function of VEGF-A. Another study, contrastingly, revealed that VEGF-C promotes the formation of functional lymphatic vessels in adults [38]. Several studies have confirmed that VEGF-C plays a crucial role in lymph angiogenesis [39,40].

### 4.8. Aging Induced Oxidative Changes in Lymphatic Vessels

Reactive oxygen species (ROS) in moderate quantities act as molecular signals and play a critical role in maintenance of vascular cell functions [41]. Damage to the endothelial cell membrane leads to microvascular changes triggered by several factors, including lipid peroxidation. ROS damages biomembranes and, consequently, causes lipid peroxidation to occur, thus influencing the integrity of the endothelium as well as the cell permeability. The interstitial accumulation of lipoperoxide (produced by the degradation of cell membranes) could have toxic effects and therefore lead to trophic changes and reductions in lymphatic contractility [42]. In 1993, it was shown that in conditions of impaired lymphatic drainage, lipoperoxides were deposited in the skin and these deposits led to alterations in the tissues (characteristic of lymphedema) [43].

Aging also produces alterations in the activity—and expression—of superoxide dismutase (SOD) in different tissues, including blood and lymphatic vessels [44]. SOD activity has been shown to be reduced in aged MLVs [44]. Reduced SOD activity could play a role in the impairment of mesenteric lymph transport function associated with aging. It is believed that reduced SOD, an antioxidant defense enzyme, can contribute to increases in ROS in older vessels and is, therefore, capable of increasing oxidative stress and, consequently, result in high oxidative damage to the tissue [42]. In contrast, in young mesenteric lymphatics, decreases in contraction frequency, lymphatic pump function and ejection fraction caused by high doses of superoxide anions were attenuated with the application of SOD [42].

### 4.9. Aging-Induced Acute Inflammation Changes and Their Effects on Lymphatic Vessels and Perilymphatic Tissues

Aging-induced changes in mast cell function influence the responses of MLVs to acute inflammation [29]. The release of histamine in the mesentery activates NF-κB signaling due to activated mast cells [29]. NF-κB is elevated in resting aged mesenteric tissues. For this reason, the initiation of an acute inflammatory response of NF-κB, and accompanying lymphatic responses that follow (e.g., the permeability of the lymphatic vessels and the trafficking of immune cells) are limited [29].

Histamine is known to play a role in the functioning of immune cells, including multidirectional crosstalk between mast cells and macrophages and the chemoattraction of several immune cells [45]. Furthermore, histamine can increase lymphatic permeability. For these reasons, it is believed that histamine has an important role in inflammatory activities in perilymphatic tissues [46]. Therefore, the functional condition of the mast cells close to the MLV plays an important role in the recruitment of eosinophils and class-II positive MHC cells to the lymphatics during acute inflammation. Aging decreases these responses during acute inflammation leading to reduction in immune cells and their efficacy in mesenteric perilymphatic tissue cells [47]. To conclude, mast cells play a central role in lymphatic inflammatory processes as they trigger NF-κB and, consequently, acute inflammatory reactions in the mesentery via histamine regulatory mechanisms.

### 4.10. Lymphatic Senescence and Immune Function

Alterations in the lymphatic system and function, as e.g., seen during aging, can accompany metabolic syndrome, obesity, cancer or infections lead to modulation of the immune response. This can occur via alterations in the transport of antigens, trafficking of antigen-presenting cells, regulation of T-cells differentiation and modulation of immunosuppressive responses. These changes can sometime evolve into a vicious cycle of events [3]. It has been shown that loss of Toll-like receptors (TLR) function results in aggravation of lymphedema, increases in fat deposition and tissue fibrosis, decreases in interstitial flow and the infiltration of macrophages. The stasis of the lymphatic fluid in lymphedema leads to an increase in the inflammatory response, often deleterious over time [2,48]. An important example we have with lymphedema patients where they often develop fungal, viral, bacterial diseases [7]. In addition, as adults age, there is a reduction of the lymphoid tissue in both cortex and medulla of the lymph nodes [49]. This loss in lymphoid tissue was also recorded in aged rats where the available data showed both a decrease in the number of lymph nodes and a histological degeneration of the aged lymph nodes [49]. These age-associated degenerations in lymphoid tissues negatively affect the efficiency of the immune system, which could potentially predispose the elderly to the development of cancer [49,50].

A notable finding from isolated MLVs in 9-month old mice was that bacteria tended to accumulate near the valves of lymphatic vessels [29]. These valve areas, even in MLVs obtained from adult mice, show a reduced investiture of muscle cells in their walls. This could lead to an increase in local permeability as well as raise the potential for an accompanying increase in the permeability to bacteria in these aged lymphatic vessels in the mesentery [29]. Furthermore, the injection of microorganisms in both young and aged mice showed a noticeable increase in bacterial accumulation only in the elderly near the tissues surrounding the lymphatic vessels [13]. These results suggest that as the lymphatic vessels age, their barrier function shows to be impaired thus promoting easier susceptibility to pathogen transport [13].

Lipopolysaccharide (LPS), an endotoxin, applied at the MLVs of cattle and rats leads to relaxation and inhibition of spontaneous phasic contractions of the MLVs [51]. Other studies on contractile function in aged MLVs reveal that LPS-induced acute peritoneal inflammation induces no changes in lymphatic tone nor in phasic lymphatic contractility [51]. The lack of responsiveness of aged MLVs to acute inflammation induced by pathogens limits their ability to respond to changes in the surrounding tissue microenvironment [51].

In the elderly, there is up to 70% reduction of circulating lymphocytes as compared to in young adults [52]. The decrease in the number of lymphocytes in the blood is generally believed to be due to a reduction in the mass of circulating T lymphocytes and/or due to a malfunction of the lymphatic vessels.

### 4.11. Lymphatic Senescence and Clinical Outlook

The main risk factor for many neurological disorders, such as Alzheimer’s disease, is aging [53]. In addition, aging has also been reported to be associated with peripheral lymphatic dysfunction [35,54]. Taken together, these observations have led to the hypothesis that poor function of the meningeal lymphatics could potentially contribute towards some aspects of cognitive decline associated with the aging process. It has been observed that meningeal dysfunction in young and adult mice leads to an alteration of the cerebrospinal fluid (CSF) as it reduces paravascular influx of macromolecules and increases in interstitial macromolecule efflux leading to learning and memory deficits. Meningeal dysfunction in elderly mice, on the other hand, may be the basis of some aspects of cognitive decline seen in these animals. Interestingly, treatment of older mice with vascular endothelial growth factor C (VEGF-C) has been shown to improve meningeal lymphatic drainage of macromolecules from the CSF as well as led to improvements in blood flow to the brain, memory and learning [55].

Recent work has shown that glymphatics (a network of vessels located in the central nervous system) is modulated by meningeal lymphatic function suggesting that there is a link between the lymphatic and glymphatic vascular systems. Aging also leads to a progressive deterioration of the cerebral vascular function, which in Alzheimer’s (AD) is aggravated by amyloid angiopathy. The malfunction of the meningeal lymphatic vessels and the flow alone, also caused by aging, is related to the reduced inflow of paravascular cerebrospinal fluid and reduced outflow of interstitial fluid. Therefore, this leads to a reduction of macromolecules solutes in the brain via the glymphatic pathway, manifesting cognitive impairment in amyloid pathology. Some data suggest that aging-induced alterations in immunity and meningeal lymphatic drainage could lead to cognitive decline [56]. It remains important to highlight the impact of the brain’s lymphatic and glymphatic vascular systems with peripheral and central immune function and its implications during aging as the glymphatic system is considered the fluid drainage system responsible for transfer of solutes (peaks also misfolded proteins) and interstitial fluids [57]. Finally, glymphatic clearance is very relevant in AD since it has been pointed out that the cerebral lymphatic vascular system with advancing age shows a reduced drainage of solutes and interstitial fluids, creating the risk of not eliminating the A𝛽 oligomers promoting deposition [57].

### 4.12. Limitations

A possible limitation could be that only “aging” instead of “ageing” was used to search for literature. From our previous experiences and publications, and to prevent confusion, we have always used one spelling. We do not believe this is a major limitation, as “aging” in American and Canadian English is the preferred spelling and moreover, because it is in major use.

## 5. Conclusions and Perspectives for Future Scientific Research

Although in recent decades, important advances have been made on the principles of physiological regulation of the transport of lymphatic fluids, we are still a long way from having a complete understanding. One of the factors that determines lymphatic function is determined by complex factors of lymphatic hydrodynamics, for example, passive and active lymphatic pumps, pressure and lengthening of the lymphatics, the consequent combination of extrinsic and intrinsic flows. Not to mention the important changes in the transport capacities and sensitivities of the flow of the lymphatic vessels largely influenced by perturbations. Finally, some normal and pathological conditions are capable of impairing the various lymphatic transport functions causing important biological and clinical problems. An example is the effect of aging on lymphatic transport. Therefore, it is essential to understand the systems that guide and regulate lymphatic transport functions, all to improve the knowledge of lymphatic dysfunction on the different pathological processes, with the aim of promoting the development of therapies to treat these processes more effectively and to counteract these dysfunctions.

## Data Availability

This work did not report any data.

## Abbreviations

AD	Alzheimer disease
CSF	Cerebrospinal fluid
ECs	Endothelial cells
eNOS	Endothelial nitric oxide synthase
ICG	Indocyanine green
ISF	Interstitial fluid
LPS	Lipopolysaccharide
MLC20	Myosin light chain (20 kDa)
MLVs	Mesenteric lymphatics vessels
NF-κB	Nuclear factor kappa-light-chain-enhancer of activated B cells
NO	Nitric oxide
RNS	Reactive nitrogen species
ROS	Reactive oxygen species
SOD	Superoxide dismutase
TLR	Toll-like receptors
UVB	Ultraviolet B light
VEGF-A	Vascular endothelial growth factor A
VEGF-C	Vascular endothelial growth factor C

## References

[1] Brix B., Apich G., Roessler A., Ure C., Schmid-Zalaudek K., Hinghofer-Szalkay H., Goswami N. (2020). Fluid shifts induced by physical therapy in lower limb lymphedema patients. J. Clin. Med..

[2] Brix B., Apich G., Ure C., Roessler A., Goswami N. (2020). Physical therapy affects endothelial function in lymphedema patients. Lymphology.

[3] Brix B., Sery O., Onorato A., Ure C., Roessler A., Goswami N. (2021). Biology of Lymphedema. Biology.

[4] Gashev A.A. (2010). Basic mechanisms controlling lymph transport in the mesenteric lymphatic net. Ann. N.Y. Acad. Sci..

[5] Zawieja D.C. (2009). Contractile physiology of lymphatics. Lymphat. Res. Biol..

[6] Dixon J.B. (2010). Lymphatic lipid transport: Sewer or subway?. Trends Endocrinol. Metab..

[7] Chakraborty S., Davis M.J., Muthuchamy M. (2015). Emerging trends in the pathophysiology of lymphatic contractile function. Semin. Cell Dev. Biol..

[8] Goswami N. (2017). Falls and fall-prevention in older persons: Geriatrics meets spaceflight!. Front. Physiol..

[9] Goswami N., Blaber A.P., Hinghofer-Szalkay H., Montani J.P. (2017). Orthostatic intolerance in older persons: Etiology and countermeasures. Front. Physiol..

[10] Boss G.R., Seegmiller J.E. (1981). Age-related physiological changes and their clinical significance. West. J. Med..

[11] Montagna W., Carlisle K. (1990). Structural changes in ageing skin. Br. J. Dermatol..

[12] Gashev A., Chatterjee V. (2013). Aged lymphatic contractility: Recent answers and new questions. Lymphat. Res. Biol..

[13] Zolla V., Nizamutdinova I.T., Scharf B., Clement C.C., Maejima D., Akl T., Nagai T., Luciani P., Leroux J.-C., Halin C. (2015). Aging-related anatomical and biochemical changes in lymphatic collectors impair lymph transport, fluid homeostasis, and pathogen clearance. Aging Cell.

[14] Ince C., De Backer D., Mayeux P.R. (2020). Microvascular dysfunction in the critically Ill. Crit. Care Clin..

[15] Turner V.M., Mabbott N.A. (2017). Structural and functional changes to lymph nodes in ageing mice. Immunology.

[16] Kajiya K., Kunstfeld R., Detmar M., Chung J.H. (2007). Reduction of lymphatic vessels in photodamaged human skin. J. Dermatol. Sci..

[17] Karaman S., Buschle D., Luciani P., Leroux J.C., Detmar M., Proulx S.T. (2015). Decline of lymphatic vessel density and function in murine skin during aging. Angiogenesis.

[18] Pan W.R., Suami H., Taylor G.I. (2008). Senile changes in human lymph nodes. Lymphat. Res. Biol..

[19] Gashev A.A., Zawieja D.C. (2010). Hydrodynamic regulation of lymphatic transport and the impact of aging. Pathophysiology.

[20] Bridenbaugh E.A., Nizamutdinova I.T., Jupiter D., Nagai T., Thangaswamy S., Chatterjee V., Gashev A.A. (2013). Lymphatic muscle cells in rat mesenteric lymphatic vessels of various ages. Lymphat. Res. Biol..

[21] Zawieja D.C., Davis K.L., Schuster R., Hinds W.M., Granger H.J. (1993). Distribution, propagation, and coordination of contractile activity in lymphatics. Am. J. Physiol..

[22] Nagai T., Bridenbaugh E.A., Gashev A.A. (2011). Aging-associated alterations in contractility of rat mesenteric lymphatic vessels. Microcirculation.

[23] Shang T., Liang J., Kapron C.M., Liu J. (2019). Pathophysiology of aged lymphatic vessels. Aging.

[24] Nizamutdinova I.T., Maejima D., Nagai T., Meininger C.J., Gashev A.A. (2017). Histamine as an endothelium-derived relaxing factor in aged mesenteric lymphatic vessels. Lymphat. Res. Biol..

[25] Ohhashi T., Kawai Y., Azuma T. (1978). The response of lymphatic smooth muscles to vasoactive substances. Pflugers Arch..

[26] Mignini F., Sabbatini M., Coppola L., Cavallotti C. (2012). Analysis of nerve supply pattern in human lymphatic vessels of young and old men. Lymphat. Res. Biol..

[27] Chakraborty S., Nepiyushchikh Z., Davis M.J., Zawieja D.C., Muthuchamy M. (2011). Substance P activates both contractile and inflammatory pathways in lymphatics through the neurokinin receptors NK1R and NK3R. Microcirculation.

[28] Henry C.B., Duling B.R. (1999). Permeation of the luminal capillary glycocalyx is determined by hyaluronan. Am. J. Physiol..

[29] Pal S., Meininger C.J., Gashev A.A. (2017). Aged lymphatic vessels and mast cells in perilymphatic tissues. Int. J. Mol. Sci..

[30] Gashev A.A., Davis M.J., Zawieja D.C. (2002). Inhibition of the active lymph pump by flow in rat mesenteric lymphatics and thoracic duct. J. Physiol..

[31] Cueni L.N., Detmar M. (2006). New insights into the molecular control of the lymphatic vascular system and its role in disease. J. Invest. Dermatol..

[32] Sawane M., Kajiya K. (2012). Ultraviolet light-induced changes of lymphatic and blood vasculature in skin and their molecular mechanisms. Exp. Dermatol..

[33] Kajiya K., Hirakawa S., Detmar M. (2006). Vascular endothelial growth factor-A mediates ultraviolet B-induced impairment of lymphatic vessel function. Am. J. Pathol..

[34] Baluk P., Fuxe J., Hashizume H., Romano T., Lashnits E., Butz S., Vestweber D., Corada M., Molendini C., Dejana E. (2007). Functionally specialized junctions between endothelial cells of lymphatic vessels. J. Exp. Med..

[35] Jakic B., Kerjaschki D., Wick G. (2020). Lymphatic capillaries in aging. Gerontology.

[36] Kidoya H., Naito H., Takakura N. (2010). Apelin induces enlarged and nonleaky blood vessels for functional recovery from ischemia. Blood.

[37] Sawane M., Kidoya H., Muramatsu F., Takakura N., Kajiya K. (2011). Apelin attenuates UVB-induced edema and inflammation by promoting vessel function. Am. J. Pathol..

[38] Breier G. (2005). Lymphangiogenesis in regenerating tissue is VEGF-C sufficient?. Circulat. Res..

[39] Breslin J.W., Gaudreault N., Watson K.D., Reynoso R., Yuan S.Y., Wu M.H. (2007). Vascular endothelial growth factor-C stimulates the lymphatic pump by a VEGF receptor-3-dependent mechanism. Am. J. Physiol. Heart Circulat. Physiol..

[40] Hartiala P., Suominen S., Suominen E., Kaartinen I., Kiiski J., Viitanen T., Alitalo K., Saarikko A.M. (2020). Phase 1 Lymfactin® study: Short-term safety of combined adenoviral VEGF-C and lymph node transfer treatment for upper extremity lymphedema. J. Plastic Reconstruct. Aest. Surg..

[41] Cai H., Li Z., Dikalov S., Holland S.M., Hwang J., Jo H., Dudley S.C., Harrison D.G. (2002). NAD(P)H oxidase-derived hydrogen peroxide mediates endothelial nitric oxide production in response to angiotensin II. J. Biol. Chem..

[42] Thangaswamy S., Bridenbaugh E.A., Gashev A.A. (2012). Evidence of increased oxidative stress in aged mesenteric lymphatic vessels. Lymphat. Res. Biol..

[43] Ohkuma M. (1993). Lipoperoxide in the dermis of patients with lymph stasis. Lymphology.

[44] Santa María C., Ayala A., Revilla E. (1996). Changes in superoxide dismutase activity in liver and lung of old rats. Free Radic. Res..

[45] O’Mahony L., Akdis M., Akdis C.A. (2011). Regulation of the immune response and inflammation by histamine and histamine receptors. J. Allergy Clin. Immunol..

[46] Sato M., Sasaki N., Ato M., Hirakawa S., Sato K., Sato K. (2015). Microcirculation-on-a-chip: A microfluidic platform for assaying blood- and lymphatic-vessel permeability. PLoS ONE.

[47] Chatterjee V., Gashev A.A. (2014). Mast cell-directed recruitment of MHC class II positive cells and eosinophils towards mesenteric lymphatic vessels in adulthood and elderly. Lymphat. Res. Biol..

[48] Ly C.L., Kataru R.P., Mehrara B.J. (2017). Inflammatory manifestations of lymphedema. Int. J. Mol. Sci..

[49] Ahmadi O., McCall J.L., Stringer M.D. (2013). Does senescence affect lymph node number and morphology? A systematic review. ANZ J. Surg..

[50] Sano S., Wang Y., Walsh K. (2018). Clonal Hematopoiesis and its impact on cardiovascular disease. Circ. J..

[51] Gasheva O., Knippa K., Muthuchamy M., Gashev A. (2009). Age-related alterations of active pumping mechanisms in rat thoracic duct. Microcirculation.

[52] Luscieti P., Hubschmid T., Cottier H., Hess M.W., Sobin L.H. (1980). Human lymph node morphology as a function of age and site. J. Clin. Pathol..

[53] Grimbaldeston M.A., Metz M., Yu M., Tsai M., Galli S.J. (2006). Effector and potential immunoregulatory roles of mast cells in IgE-associated acquired immune responses. Curr. Opin. Immunol..

[54] Shakoory B., Fitzgerald S.M., Lee S.A., Chi D.S., Krishnaswamy G. (2004). The role of human mast cell-derived cytokines in eosinophil biology. J. Interferon. Cytokine Res..

[55] Da Mesquita S., Louveau A., Vaccari A., Smirnov I., Cornelison R.C., Kingsmore K.M., Contarino C., Onengut-Gumuscu S., Farber E., Raper D. (2018). Functional aspects of meningeal lymphatics in ageing and Alzheimer’s disease. Nature.

[56] Da Mesquita S., Fu Z., Kipnis J. (2018). The meningeal lymphatic system: A new player in neurophysiology. Neuron.

[57] Santoro A., Spinelli C.C., Martucciello S., Nori S.L., Capunzo M., Puca A.A., Ciaglia E. (2018). Innate immunity and cellular senescence: The good and the bad in the developmental and aged brain. J. Leukoc. Biol..

